# Notices

**Published:** 1867-09

**Authors:** 


### NOTICES.

---

A Family Paper. — There is no weekly newspaper coming to our table more worthy of general patronage among all classes of christian men than The Watchman & Reflector, 161 Washington Street, Boston, Mass., $3 a year, by Ford & Olmstead. It is now in its 48th year; eight pages, large clear type and good paper. As an evidence of its interesting character we give the subjects treated of in one of the eight pages. The Invention of Printing; Unitarianism and Othodoxy; Hugh Miller the Philosopher and John Holm the Mechanic; Frightened into Religion; Paul's Quotation from Greek; Human Motives and Estimates; Paradise Music; Led by Providence; Some other Name; Sense of Housekeepers; Worth Hearing; Go to Work; Death at the Breakfast-table; Mr. Beecher's Preaching; Dribble Drone's Goose; A Lost Love.

The New England Farmer Monthly, 8vo, published at 34 Merchants Row, Boston, R. P. Eaton & Co., $1 50 a year, has in its May issue over sixty different articles of practical value to every farmer. Premiums for obtaining from two to eighty-four subscribers are offered, ranging from two to a hundred and twenty-nine dollars. Specimens sent post-paid for twenty cents.

The Missionery Herald, in its 63d volume, is published by the American Board of Commissioners for Foreign Missions at the Missionery House, 33 Pemberton Square, Boston. The May number contains a biographical sketch of Rev. Wm. Goodel, who died at the house of his son in Philadelphia, Feb. 18, 1867. When a baby he seemed to have so frail a constitution that a relative inquired in disgust if he was worth raising. He was frail all his days, yet lived to a good old age and worked hard to the last, and worked to purpose, too; few men of modern times have done as much. His history illustrates a frequently observed fact, that frail persons often outlive the more robust by half a century simply because they are compelled to a carefulness of their health which the strong and hearty can not be made to believe is at all necessary, teaching the great practical law, it is worth while to take care of the health.

JUST published in royal 8vo, fine laid paper, 432 pages, price $3, THE CHRIST OF THE APOSTLES' CREED: The Voice of the Church against Arianism, Strauss and Renan, with an Appendix by Rev. W. A. Scott, D. D., Pastor of the Forty-second Street Presbyterian Church. A. D. F. Randolph, New York. Also, in press and soon to appear by the same author, the second edition, THE CENTURIONS OF THE GOSPEL; With Discourses on "The Choice of a Profession." "Our Responsibility for our Fellow-men," and "The Piety and Patriotism of Praying for our Rulers." Price $2 00.

Other works by the same author:

THE WEDGE OF GOLD; or Achan in El Dorado. Published in San Francisco, and also by the Presbyterian Board in Philadelphia. Price, 60 cents. TRADE AND LETTERS; Their Journeyings round the World. Delivered before, and Published at the request of, the "Mercantile Library Association of San Francisco." Price 90 cents. THE GIANT JUDGE; or, Samson the Hebrew Hercules. Published in San Francisco, and also by the Presbyterian Board in Philadelphia. 85 cents. MOSES AND THE PENTATEUCH. A reply to Bishop Colenso. London: Freeman, 102 Fleet St. $1 50. DANIEL. A Model for Young Men. $1 50. These books can be had of Anson D. F. Randolph, 770 Broadway, New York, who will forward copies by mail prepaid, on receipt of the price.

CULTIVATE THE BEAUTIFUL AND PROGRESSIVE. — The HOME JOURNAL enlarged, embellished and enriched. Says the New York Commercial Advertiser, "Under its present management will have a new lease of prosperity and popularity." Edited by George Perry and J. B. Elliot. Of the former, the American Art Journal says, "He is a terse, vigorous and elegant writer, varied in his style, but always eminently readable, and is emphatically the right man in the right place." Mr. E. was a special favorite of N. P. Willis, and his contributions stand high in the literary world; while the first dawnings of the new youth of the Home Journal come through the tireless energies and good judgement of Morris Phillips, who has the business management of the paper.

THE AMERICAN TRACT SOCIETY, 150 Nassau Street, New York, have issued a Catalogue of Volumes and Libraries, adapted to Pastors, Families, Bible-classes and Sabbath schools—with the prices of many single volumes, and also the cost of well selected Libraries from Ten Dollars and upwards. This catalogue will be sent, post-paid, for five cents by addressing S. W. Stebbins, Depository, 150 Nassau Street, New York, or will be given to all who apply for it personly, with a view to purchase; it with the Society's other publications, may be had at 40 Cornhill, Boston, 75 State Sreet, Rochester, N. Y.; at Philadelphia, 1210 Chestnut Street; Richmond, Va., 131 Broad Street; Cincinnati, O., Walnut Street, near Fourth, Seely Wood, Agent; Baltimore, Md., 73 West Fayette Street; St. Louis, Mo., 9 South Fifth St: Chicago, Ill., 7 Custom House Place. Our readers would do well to bear this directory in mind. The publications of this Society are numerous, and useful both to individuals and to families and are much cheaper in proportion than the publications of any booksellers in the United States. The Family Christian Almanac is one of the Society's issues and is worthy of a place in every family, not only for its correctness, but for the large amount of useful reading it contains, for only ten cents; sixty pages besides an inviting cover. The Reign of Grace, by Thomas Chalmbers, D. D.; Pastoral Reminiscences, by the late Rev. Martin Moore. of Boston. 64 pps. Phil. Kennedy, by H. N. N. 128 pages; Hours with Mamma, by Mrs. S. E. Dawes, 306 pages. "The Climbers;" a beautiful story of the good Providence of God, 268 pages, with illustrations, are among the late noticeable issues of this veteran Society, whose glorious work grows and widens with increasing years, and long may it do so.

"The Redeemer;" being a sketch of the history of the Redemption, by Edmund de Pressense, translated from the second edition by Rev. J. H. Myers, D. D., published by the American Tract Society, 28 Cornhill, Boston, and 13 Bible House, New York, by Broughton and Wyman. 412 pages, 12mo, price $2 by mail. A most valuable publication for all christian hearts.

